# Tripartite motif–containing 9 promoted proliferation and migration of bladder cancer cells through CEACAM6-Smad2/3 axis

**DOI:** 10.1007/s12079-023-00766-7

**Published:** 2023-05-30

**Authors:** Zhao-Cun Zhang, Hai-Feng Zhao, Zhuang Sun, Yi Li, Ming-Lei Zhong, Bao-Hai Wang, Xian-Zhou Jiang

**Affiliations:** 1 Department of Urology, Qilu Hospital of Shandong University, Jinan, Shandong Province China; 2 Department of Urology, Liangshan People’s Hospital, Jining, Shandong Province China

**Keywords:** TRIM9, CEACAM9, Smad2/3, Bladder cancer

## Abstract

Studies have shown that tripartite motif–containing (TRIM) family proteins function as E3 ubiquitin ligases and play essential roles in cancer biology. In the present study, we validated a contribution of TRIM9 to bladder cancer progression. 296 patients derived from The Cancer Genome Atlas (TCGA) database and 22 clinical specimens were included, in which accumulated TRIM9 correlated with the poor prognosis and higher relapse in bladder patients. In vitro, TRIM9 promoted bladder cancer cells Biu-87 and T24 cell proliferation and migration. Meanwhile, overexpression of TRIM9 reduced the chemosensitivity in Biu-87 and T24 to mitomycin C (MMC) and gemcitabine (GEM). As an underlying mechanism, we found that TRIM9 stimulated carcinoembryonic antigen 6 (CEACAM6) upregulation, which further facilitated Smad2/3-matrix metalloproteinase 2 (MMP2) signaling activation both in vitro and in vivo. Those results indicated that TRIM9 facilitated bladder cancer development and chemoresistance by CEACAM6-Smad2/3 axis. TRIM9 and its associated molecules could be a potential diagnostic indicator and therapeutic target in bladder cancer.

**Graphic Abstract:**

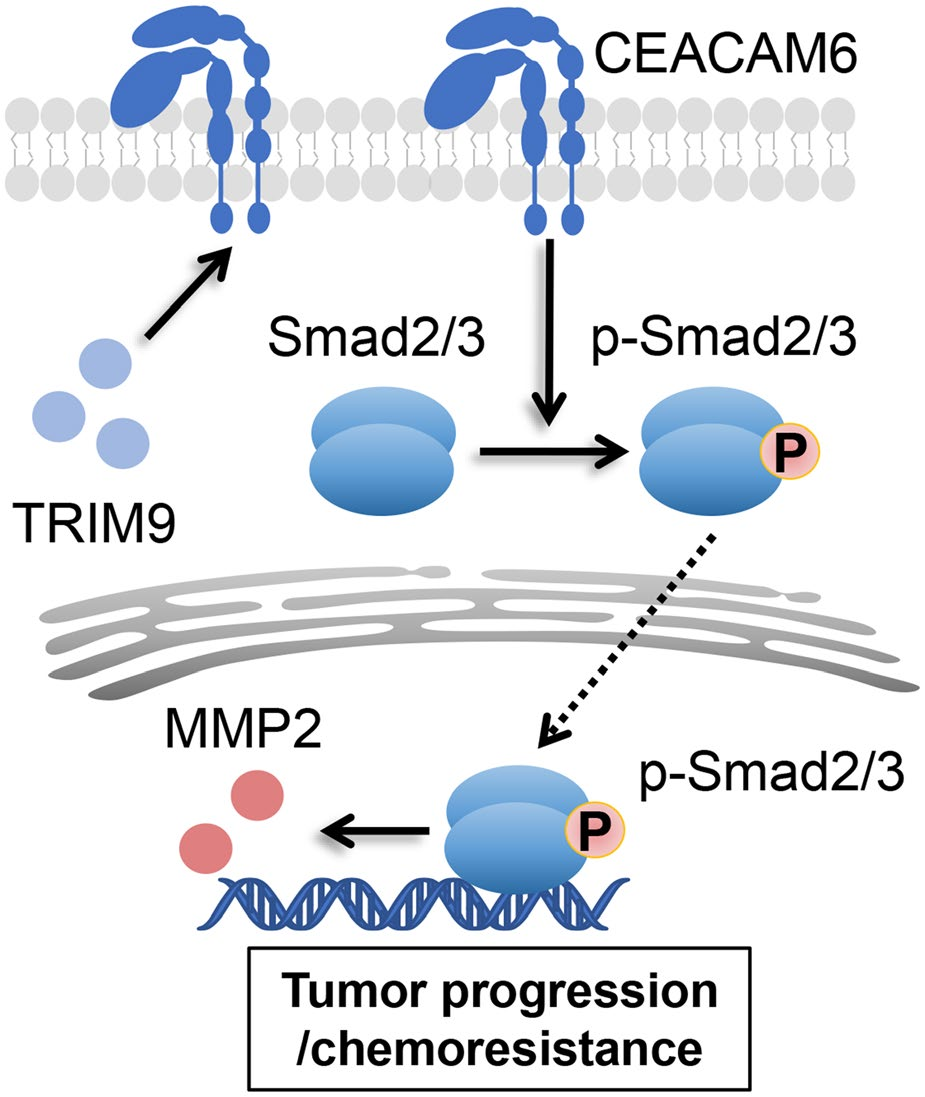

**Supplementary Information:**

The online version contains supplementary material available at 10.1007/s12079-023-00766-7.

## Introduction

Bladder cancer is among the most prevalent and aggressive cancers worldwide, with approximately 573,000 new cases and 213,000 deaths reported in 2020 (Sung et al. 2021). This disease is a heterogeneous epithelial malignancy that present most commonly as non-muscle invasive. However, about 25% of patients have muscle-invasive or metastatic disease at the time of initial diagnosis, which portended a worse prognosis (Robertson et al. 2017). Remarkable advances in early diagnosis and treatment have been achieved (Wu et al. 2020; Wong et al. 2017), but the high recurrent rate, mortality and poor prognosis are still main problems in bladder patients. Even after radical cystectomy and recommended drug treatments, a number of patients experience primary invasion and distant metastasis (Lopez-Beltran et al. 2021). Therefore, there is a severe urgent to understand the potential molecular mechanisms and explore new therapeutic targets for this disease.

TRIM9 belongs to the tripartite motif containing (TRIM) family which has been identified as a subfamily of the RING type E3 ubiquitin ligase and was implicated in a variety of cellular functions and biological process, including regulation of cellular proliferation, division, apoptosis and development processes (Venuto et al. 2019; Chen et al. 2012). The TRIM family consists of over 70 members, and several TRIM proteins, such as TRIM8, 13,19,24,27,29,31, 32, 33, 40,59 and 69, have recently been verified to be involved in oncogenesis or tumor progression by regulating specific signal pathways, such as p53, RARα, NF-κB, in various kinds of cancers (Hatakeyama 2011). Specifically for bladder cancers, TRIM 26,59 and 65 have been shown to enhance cellular invasion, migration and epithelial-to-mesenchymal (EMT) and support cell aggressiveness in bladder carcinoma development (Xie et al. 2021; Chen et al. 2017; Wei et al. 2018). TRIM9 protein is known as a brain-specific E3 ligase and contributes to neuronal axon growth in the brain and associated with several neurological disorders (Tanji et al. 2010). Moreover, methylated TRIM9 was reported to be abnormally expressed in breast tumor tissues and plasma in metastatic breast cancer patients, and can serve as a novel blood biomarker in breast cancer (Mishima et al. 2015). More recently, TRIM9 overexpression was shown to promote uterine leiomyoma development through NF-κB signaling pathway (Yang et al. 2020). However, little is known about the expression profile of TRIM9 in bladder cancers, and its biological role in this disease remains unclear.

In the present study, we investigated the underlying association between TRIM9 expression and the development of bladder cancer in vitro and in vivo. We initially determined that the upregulated TRIM9 was closely correlated to the clinical stages, tumor recurrence and poor survival in bladder cancer patients. Mechanically, we further demonstrated that the overexpressed TRIM9 increased tumor cells proliferation, migration and drug resistance via CEACAM6-Smad2/3 signaling activation in bladder cancer. Our evidences highlighted the critical role of TRIM9 in bladder cancer progression and provided promising therapeutic targets for clinical treatment.

## Materials and methods

### Cell culture and reagents

Human bladder cancer cell lines Biu-87 and T24 were obtained from American Type Culture Collection and maintained in Roswell Park Memorial Institute 1640 culture medium containing 10% fetal bovine serum in a 5% CO_2_ incubator at 37℃. TRIM9 overexpressed Biu-87 and T24 cell lines were established by SLPLAB (China). Chemotherapeutic MMC and GEM were purchased from Sigma-Aldrich (USA). Smad2/3 inhibitor ITD-1 was purchased from Selleck (USA).

### Clinical specimens and TCGA analysis

Paraffin sections of 22 human bladder tumor tissues were obtained from Department of Urology, Qilu Hospital of Shandong University, and divided into recurrent (n = 11) and non-recurrent (n = 11) groups according to 5-years follow up visit. The information of 22 patients was shown in supplementary Fig. 1. All experiments were performed according to the Declaration of Helsinki and granted by the Ethics Committee of the Qilu Hospital of Shandong University (Approval No. 2022-028). Transcript profile and survival information of 296 patients were obtained from https://www.cbioportal.org/, and analyzed by R version 4.2.0 (USA).

### Cell proliferation assay

Cell proliferation was determined by Cell Counting Kit-8 (CCK8, Solarbio, China). Biu-87 or T24 cells were seeded into a 96-well plate (1 × 10^3^ cells/well). After culture of 0, 24, 48 and 72 h, samples were incubated with 10% CCK-8 solution for 4 h. The absorbance at 450 nm was determined by microplate reader (Thermo Fisher, USA).

### Tranwell assay

Biu-87 and T24 cells were seeded in a 24-well Boyden Chamber (1 × 10^5^ cells/well, 8 μm, Corning, USA). After 24 h, cells that migrated to the underside of the membranes of each insert were stained with crystal violet, and counted under a low magnification microscope (Leica, Germany).

### RNA interference

Small interfering RNA (siRNA) against CEACAM6 were synthesized by SLPLAB (China). The sequences of siRNA for CEACAM6 are 5′-GAAATACAGAACCCAGCGAGTGC-3′ (siRNA#1) and 5′-CCGGACAGUUCCAUGUAUA-dTT-3′ (siRNA#2). Biu-87 and T24 cells were seeded in 6-well plates. When the cell density reached 60%, transient transfection was performed according to manufacturer’s instructions by importing siRNA into cells.

### Western blotting

Biu-87 and T24 cells were lysed in the RIPA lysis buffer (Solarbio, China). 25 µg protein was separated by SDS-PAGE and transferred to PVDF membranes. Then, the membranes were incubated with primary antibodies: anti-TRIM9 (PA5-100120; Thermo Fisher), anti-CEACAM6 (MA5-29144; Thermo Fisher), phosphorylated Smad2/3 (ab272332; Abcam, UK) and total Smad2/3 (ab202445; Abcam, UK) at 4ºC overnight. The membranes were incubated with HRP-conjugated secondary antibody (Thermo Fisher, USA) at room temperature and the ECL detection kit (Thermo Fisher, USA) was employed to detect the specific proteins.

### Immunostaining

Sections of tumor tissues were processed by deparaffinization, rehydration and antigen retrieval. Samples were then blocked by 5% bovine serum albumin and incubated with the following primary antibodies: phosphorylated Smad2/3 (ab272332; Abcam, UK), anti-TRIM9 (PA5-100120; Thermo Fisher) and anti-CEACAM6 (MA5-29144; Thermo Fisher) at 4ºC overnight. Samples were incubated with HRP-conjugated secondary antibody (Thermo Fisher, USA) at room temperature for 1 h. Cell nuclei were counterstained with hematoxylin for immunohistochemistry and HRP chromogenic agent/DAPI for immunofluorescence. Expression intensity was evaluated by Image Pro Plus software (USA).

### Enzyme linked immunosorbent assay (ELISA)

1 × 10^5^ Biu-87 or T24 cells seeded in 6-well plate containing 2 ml culture medium. MMP2 and MMP9 in supernatant was then quantified by human MMP2 assay kit (Beyotime, USA) and human MMP9 assay kit (Beyotime, USA) according to the protocols. Each experiment was performed for at least three independent times.

### Cell apoptosis analysis

The effect of chemotherapeutic agents (MMC and GEM) on T24 and Biu-87 cells was determined by flow cytometry. Briefly, overexpressed or siRNA treated T24/Biu-87 cells were treated with MMC (0.5 µg/ml) or GEM (0.5 µg/ml) for 48 h. After treatment, tumor cells were harvested and resuspended with 200 ml binding solution, followed with FITC-Annexin V and propidium iodide staining at 37 °C for 20 min. Then the samples were analyzed by a flow cytometry (BD Corporation, NJ, USA).

### Animal protocols

Female NOD-SCID mice (6 ~ 8 weeks) were purchased from Huafukang (Beijing, China) and raised in a specific pathogen-free facility. For tumor volume and survival assay, 2 × 10^6^ vector or TRIM9 overexpressed T24 cells were subcutaneously injected into mice (n = 6 per group). On day 25, mice were sacrificed for protein level assay. Tumor volume and survival were recorded daily. For tumor suppressive assay, 2 × 10^6^ TRIM9 overexpressed T24 cells were subcutaneously injected into mice (n = 6 per group). Mice were treated with PBS, ITD-1 (2 mg/kg), MMC (5 mg/kg) or combination on day 12, 15 and 18 by tail vein injection. Tumor volume and survival were recorded daily. The calculation formula of tumor volume: tumor volume = length × width ^2^/2. All animal experiments were performed according to the guidelines of the Ethics Committee of Qilu Hospital of Shandong University.

### Statistical analysis

Data was presented as mean ± SD and analyzed using Graphpad 7.0 statistical software. The differences between two groups were compared using an independent sample t test. Comparisons among multiple groups were analyzed using one-way ANOVA, followed by Tukey’s post hoc test. The Kaplan-Meier estimator was conducted to evaluate the overall survival. Each experiment was performed for at least three independent times. P value < 0.05 was considered statistically significant.

## Results

### TRIM9 promoted bladder cancer development and reduced chemo-sensitivity

**Fig. 1:**
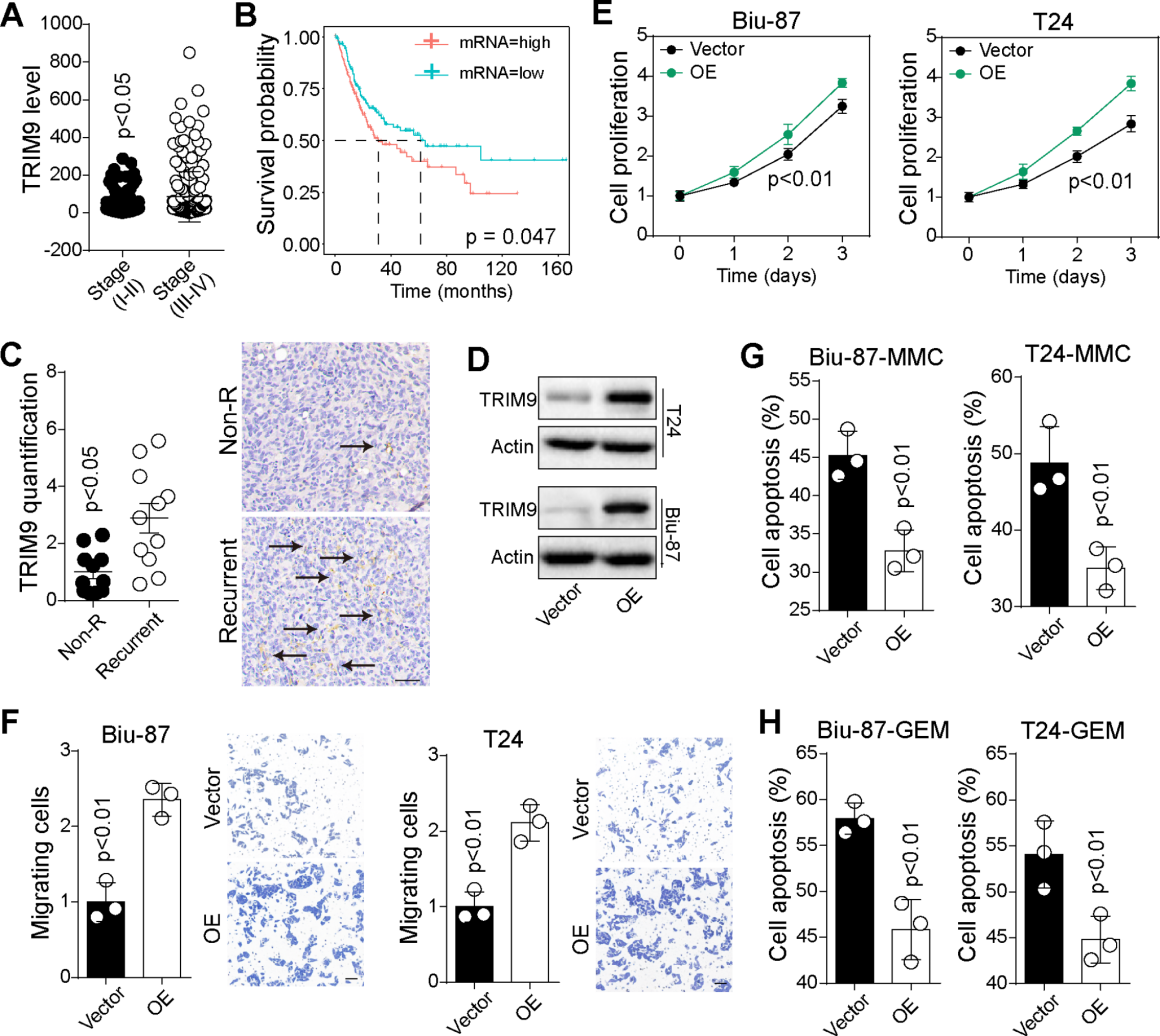
TRIM9 promoted bladder cancer development. A, mRNA expression of TRIM9 in 296 bladder cancer patients divided into high-stage (stage I ~ II) and low-stage (stage III ~ IV) groups, derived from TCGA database. B, Kaplan-Meier overall survival curve was shown according to high and low expression of TRIM9 in 296 bladder cancer patients derived from TCGA data. C, immunostaining of TRIM9 in tumor tissues from clinical non-recurrent (n = 11) and recurrent (n = 11) bladder cancer patients. The scale bar was 100 μm. Protein expression quantification was performed by Image Pro Plus software. D, western blotting of TRIM9 in vector or TRIM9 overexpressed T24/Biu-87 cells. E, cell proliferation of vector or TRIM9 overexpressed T24/Biu-87 cells. F, cell migration of vector or TRIM9 overexpressed T24/Biu-87 cells, determined by Transwell assay. The scale bar was 50 μm. G, cell apoptosis of vector or TRIM9 overexpressed T24/Biu-87 cells treated with MMC (0.5 µg/ml, 48 h). H, cell apoptosis of vector or TRIM9 overexpressed T24/Biu-87 cells treated with GEM (0.5 µg/ml, 48 h). Values are represented in mean ± SD.

To identify the role of TRIM9 during bladder cancer progression, we first analyzed its transcriptome expression in 296 bladder tumor tissues by utilizing TCGA database. The findings suggested that TRIM9 was upregulated in bladder cancer patients with high clinical stages (stage III ~ IV), when compared to those from low clinical stages (stage I ~ II, Fig. 1A). We wondered whether elevated TRIM9 influenced overall survival of bladder cancer patients. Thus, patients were divided into high (n = 148) and low (n = 148) TRIM9 expression groups. Accordingly, TRIM9-high patients had an obviously shorter survival time than patients with low TRIM9 expression (Fig. 1B). Subsequently, to further validate the role of TRIM9 in tumor recurrence, we obtained 22 clinical bladder tumor tissues, in which patients were divided into recurrent and non-recurrent groups according to 5-years follow-up visit (Fig. S1A). Immunostaining indicated that recurrent bladder cancer patients displayed a prominent increase in TRIM9 expression in comparison with non-recurrent group (Fig. 1C). Those results suggested that TRIM9 correlated with the poor prognosis in bladder cancer patients. Next, to evaluate the influence of TRIM9 on cell proliferation/migration in vitro, we established TRIM9 overexpressed T24 and Biu-87 cell lines (Fig. 1D) and conducted cell proliferation and Transwell assay. TRIM9 overexpression promoted bladder cell proliferation (Fig. 1E) and migration (Fig. 1F) in vitro. Intriguingly, TRIM9 overexpressed T24/Biu-87 cells also exhibited reduced sensitivity to chemotherapeutic drugs (MMC, Fig. 1G and GEM, Fig. 1H). Together, those results indicated that TRIM9 played an oncogenic role in promoting bladder cancer development.

### TRIM9 facilitated tumor progression through CEACAM6

**Fig. 2:**
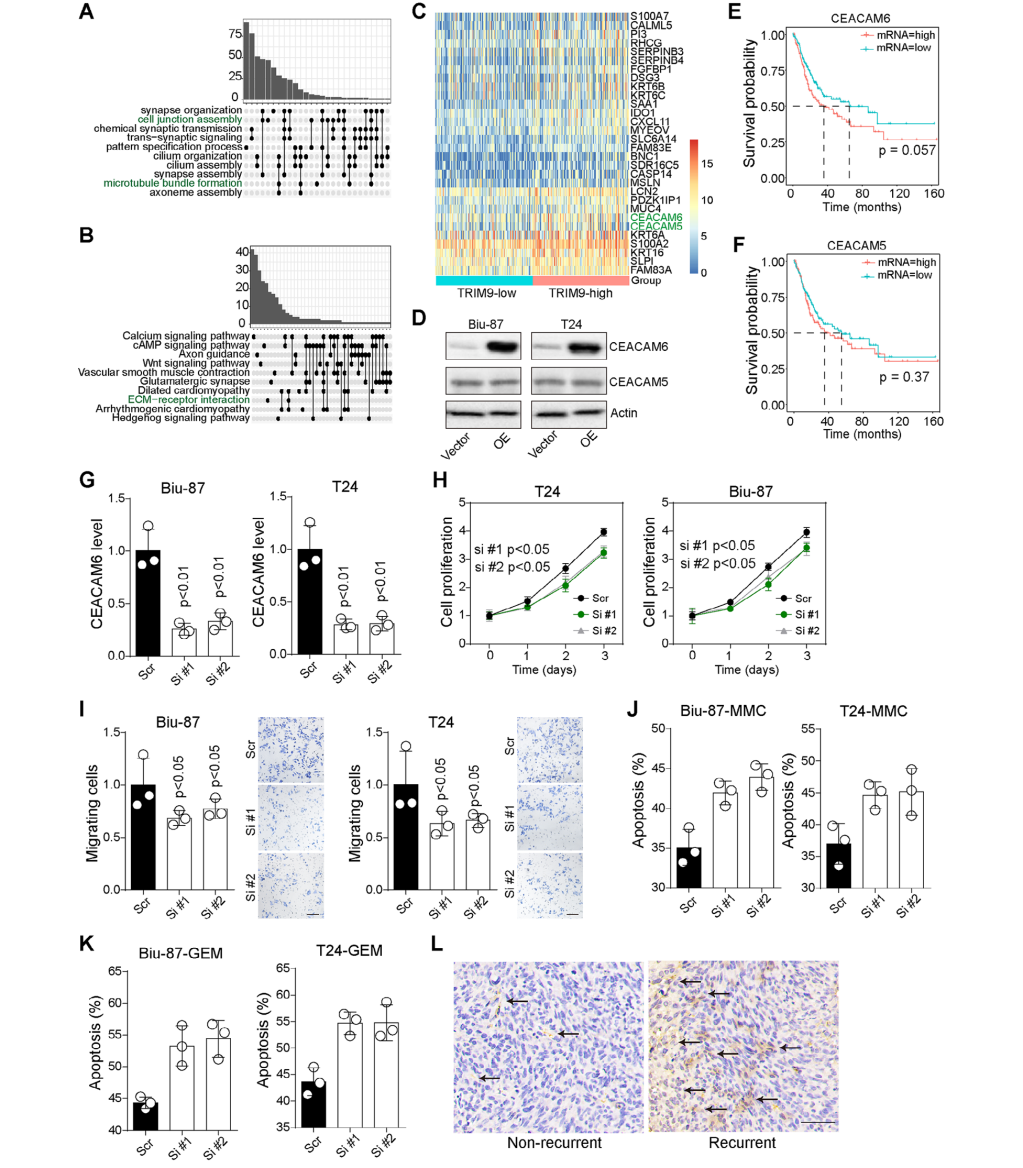
TRIM9 facilitated tumor progression through CEACAM6. A and B, GO (A) and KEGG (B) enrichment assay of 296 bladder cancer patients divided into high and low TRIM9 expression groups, derived from TCGA database. C, the top 30 upregulated genes in bladder cancer patients (high TRIM9) in comparison with low TRIM9 group. D, western blotting of CEACAM5 and CEACAM6 in vector or TRIM9 overexpressed T24/Biu-87 cells. E and F, Kaplan-Meier overall survival curve was shown according to high and low expression of CEACAM6 (E) or CEACAM5 (F) in 296 bladder cancer patients derived from TCGA data. G, mRNA expression of CEACAM6 in TRIM9 overexpressed T24/Biu-87 cells treated with scramble or CEACAM6 siRNA. H, cell proliferation of TRIM9 overexpressed T24/Biu-87 cells treated with scramble or CEACAM6 siRNA. I, cell migration of T24/Biu-87 cells treated with scramble or CEACAM6 siRNA, determined by Transwell assay. The scale bar was 50 μm. J and K, TRIM9 overexpressed T24/Biu-87 cells were treated with scramble or CEACAM6 siRNA. Then cells were treated with MMC (0.5 µg/ml, 48 h, J) and GEM (0.5 µg/ml, 48 h, K), and cell apoptosis was determined. L, immunostaining of CEACAM6 in tumor tissues from clinical non-recurrent (n = 11) and recurrent (n = 11) bladder cancer patients. The scale bar was 100 μm. Values are represented in mean ± SD.

In attempt to explore the molecular mechanism driven by TRIM9 for promoting bladder cancer development, we further evaluated the transcriptome expression of TRIM9-high patients (n = 148) compared to TRIM-low group (n = 148). Differentially expressed genes were identified, and TRIM9-involved major signaling pathways were determined by Gene Ontology (GO) and Kyoto Encyclopedia of Genes and Genomes (KEGG) enrichment (Fig. 2A and B). Notably, TRIM9 was tightly involved in cell adhesion process (cell junction assembly, microtubule bundle formation and ECM-receptor interaction). Meanwhile, the carcinoembryonic antigen-related adhesion molecules CEACAM5 and CEACAM6 were upregulated in TRIM9-high patients, when compared to TRIM-low group (Fig. 2C). Those results indicated that TRIM9 might mediate bladder cancer development through a CEACAMs dependent manner. Therefore, to clarify the role of CEACAMs, the protein level of CEACAM5 and CEACAM6 in TRIM9 overexpressed T24/Biu-87 was determined by western blotting. And elevated expression of CEACAM6 was found in TRIM9 overexpressed groups (Fig. 2D). Moreover, utilizing analyzing TCGA database, patients with high CEACAM6 expression exhibited a trend of shortened overall survival time in comparison with low CEACAM6 group (p = 0.057, Fig. 2E), whereas limited influence of CEACAM5 expression on overall survival was found (Fig. 2F). To further investigate the role of CEACAM6, CEACAM6 expression in bladder cancer cells was inhibited by siRNA (Fig. 2G). CEACAM6 deficiency efficiently suppressed cell proliferation (Fig. 2H) and migration (Fig. 2I) induced by TRIM9. Additionally, silence of CEACAM6 increased chem-sensitivity to MMC (Fig. 2J) and GEM (Fig. 2K) in TRIM9 overexpressed T24 and Biu-87 cells. Importantly, immunostaining results further confirmed elevated expression of CEACAM6 in recurrent bladder cancer patients (Fig. 2L). Collectively, those results suggested that TRIM9 mediated bladder cancer progression through CEACAM6.

### TRIM9/CEACAM6 mediated Smad2/3 signaling activation

**Fig. 3:**
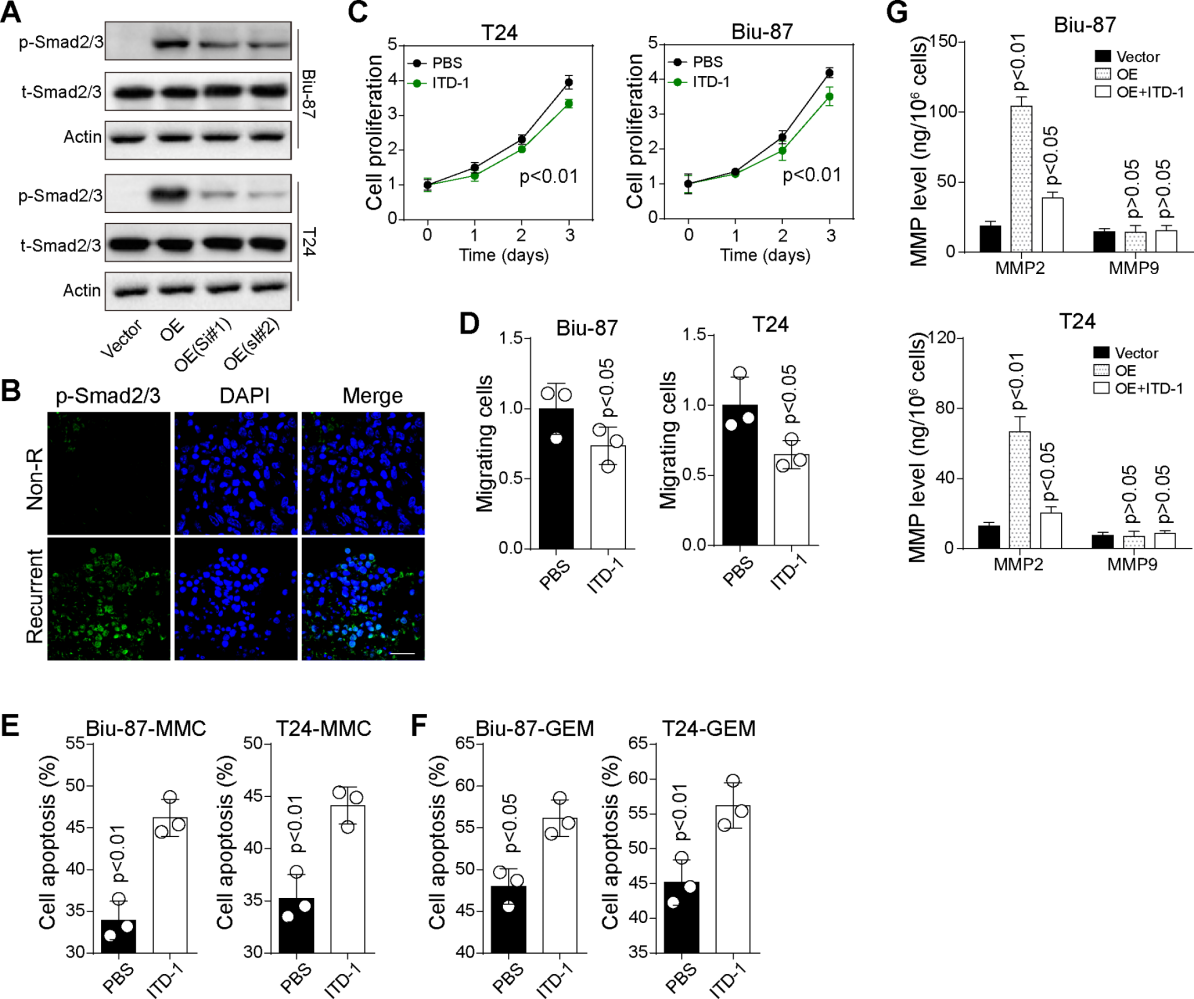
TRIM9/CEACAM6 mediated Smad2/3 signaling activation. A, western blotting of phosphorylated and total Smad2/3 in vector or TRIM9 overexpressed T24/Biu-87 cells, treated with CEACAM6 siRNA or not. B, immunofluorescence of phosphorylated Smad2/3 in tumor tissues from clinical non-recurrent (n = 11) and recurrent (n = 11) bladder cancer patients. The scale bar was 100 μm. C, cell proliferation of TRIM9 overexpressed T24/Biu-87 cells, treated with PBS or ITD-1 (30 nM). D, cell migration of TRIM9 overexpressed T24/Biu-87 cells treated with PBS or ITD-1 (30 nM), determined by Transwell assay. E and F, TRIM9 overexpressed T24/Biu-87 cells treated with PBS or ITD-1 (30 nM). Then cells were treated with MMC (0.5 µg/ml, 48 h, E) and GEM (0.5 µg/ml, 48 h, F), and cell apoptosis was determined. G, MMP2 and MMP9 quantification in supernatant from vector and TRIM9 overexpressed T24/Biu-87 cells (co-cultured with PBS or 30 nM ITD-1). Values are represented in mean ± SD.

As previously reported, CEACAMs are tightly involved in the activation of TGF-β1/Smad signaling pathway (Jensen-Jarolim et al. 2015). And Smad2/3 linker phosphorylation is an essential marker of cancer stem cells and correlates with chemoresistance development in several tumor types (Suzuki et al. 2015; Li et al. 2021). To validate the role of Smad2/3 signaling in TRIM9/CEACAM6-induced tumor progression, the expression of Smad2/3 was examined in TRIM9 overexpressed cells by western blotting. We found that overexpression of TRIM9 induced obvious upregulation of phosphorylated Smad2/3 in Biu-87 and T24 cells, and CEACAM6 silence suppressed Smad2/3 activation (Fig. 3A). Moreover, immunostaining analysis further demonstrated that recurrent patients exhibited enhanced Smad2/3 expression in tumor tissues, when compared to non-recurrent patients (Fig. 3B), indicating the potential role of Smad2/3 in TRIM9-assocaited tumor progression. Next, we treated TRIM9 overexpressed cells with Smad2/3 inhibitor ITD-1 (Fig. S1B), and cell proliferation/migration was then evaluated. As excepted, ITD-1 suppressed the cell proliferation (Fig. 3C) and migration (Fig. 3D) induced by TRIM9. Moreover, blockade of Smad2/3 signaling increased chemo-sensitivity to MMC and GEM in TRIM9 overexpressed cells (Fig. 3E and F), indicating that TRIM9 upregulated Smad2/3 to facilitate bladder cancer development and chemoresistance. Increasing evidence has suggested that Smad2/3 signaling regulates the basal and TGF-β-induced MMPs expression to facilitate tumor progression (Zare et al. 2020). Thus, supernatant of vector or TRIM9 overexpressed Biu-87/T24 cells was collected and MMP2/9 was quantified by ELISA. We found that the protein level of MMP9 was unchanged, while TRIM9 overexpression facilitated MMP2 production in Biu-87/T24 cells, and blockade of Smad2/3 signaling suppressed MMP2 expression (Fig. 3G), indicating that Smad2/3 mediated downstream MMP2 production in bladder cancer cells. Together, those data indicated that TRIM9/CEACAM6 mediated Smad2/3 signaling activation to modulate bladder cancer progression.

### TRIM9 stimulated bladder cancer growth in vivo

**Fig. 4:**
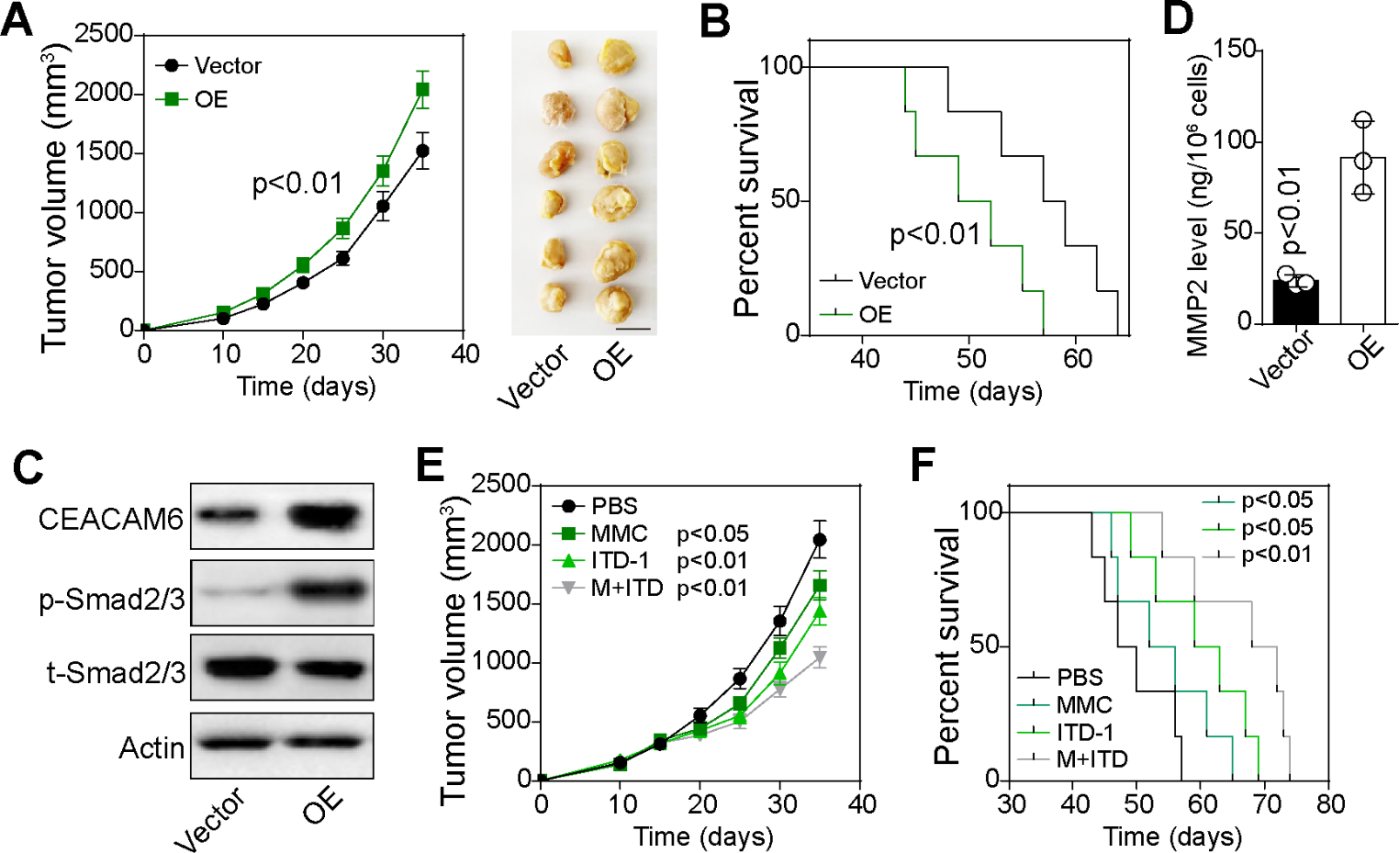
TRIM9 stimulated bladder cancer growth *in vivo.* A, tumor volume of vector or TRIM9 overexpressed T24-bearing mice (n = 6). The scale bar was 1 cm. B, overall survival of vector or TRIM9 overexpressed T24-bearing mice (n = 6). C and D, Tumor tissues in (A) were isolated on day 25. MMP2 level was detected by ELISA. The protein level of CEACAM6, phosphorylated Smad2/3 and total Smad2/3 was detected by western blotting. E and F, TRIM9 overexpressed T24-bearing mice were treated with PBS, MMC, ITB-1 and combination. Tumor volume (E) and survival time (F) were recorded. Values are represented in mean ± SD.

Motivated by our previous evidence that TRIM9 promoted CEACAM6-Smad2/3 signaling to facilitate bladder cancer cell proliferation/migration, we next sought to evaluated the influence of TRIM9 on tumor growth in vivo. To do this, 2 × 10^6^ vector or TRIM9 overexpressed T24 cells were subcutaneously injected into immunodeficient mouse. We found that TRIM9 overexpressed cells formed more rapidly growing tumors in comparison with vector cells (Fig. 4A). And TRIM9 overexpressed T24-bearing mice exhibited a shorter survival time compared to vector group (Fig. 4B), indicating that TRIM9 promoted bladder cancer development and resulted in poor prognosis in vivo. In line with the high expression of TRIM9, the protein level of CEACAM6, Smad2/3 and MMP2 were upregulated in TRIM9 overexpressed tumor tissues (Fig. 4C and D). Given the essential role of Smad2/3 signaling in TRIM9-induced tumor progression, it should be feasible to suppress Smad2/3 molecule to improve the outcome of chemotherapy in TRIM9-high patients. Thus, we combined chemotherapeutic MMC with Smad2/3 inhibitor ITD-1 for target therapy in TRIM9-overexpressed tumor model. TRIM9 overexpressed T24-bearing mice were treated with MMC, ITD-1 or combination. Intriguingly, limited tumor suppressive effects were observed in MMC treated group, which was in line with our in vitro results that TRIM9 reduced chemo-sensitivity. However, ITD-1 efficiently improved the outcome of chemotherapy (Fig. 4E) and prolonged the survival time of tumor-bearing mice (Fig. 4F). Together, our experiments provided evidence that TRIM9 plays an essential role in promoting tumor progression, and described innovative target for bladder cancer therapy.

## Discussion

Accumulating evidences have suggested that members of the TRIM family play important roles in tumor development and progression. Recent researches have reported that several TRIM proteins were involved in the regulation of cancer stem cell self-renewal by activating core transcription factors, specific signaling pathways, EMT program (Jaworska et al. 2020). For instance, TRIM28 promotes Nanog, Sox2, and Oct-4 expression (Samudyata et al. 2019; Seki et al. 2010), and TRIM27 induces EMT process and activates Akt signaling (Zhang et al. 2018). Moreover, increasing evidences indicated that TRIM proteins expression predicts survival and exerts as biomarkers in several cancers, including lung cancer, colorectal cancer and breast cancer (Zhan and Zhang 2021; Fitzgerald et al. 2013; Kawabata et al. 2017). Methylated TRIM9, which suppressed the TRIM9 mRNA expression, was reported to serve as a blood biomarker in breast cancer patients (Mishima et al. 2015). Meanwhile, TRIM9 overexpression was reported to promotes uterine leiomyoma progression via enhancement of cell proliferation, reduction of cell apoptosis and regulation of cell cycle and nuclear NF-κB expression (Yang et al. 2020). Consistent with prior evidences, the present study initially clarified a pro-tumor function of TRIM9 in bladder cancer development. Aberrant high expression of TRIM9 in bladder cancer correlates to poor survival and is an independent prognostic factor associated with a higher risk of relapse in bladder patients. Furthermore, we demonstrated that TRIM9 promoted bladder cancer progression and chemoresistance through the regulation of cell proliferation, apoptosis, migration via CEACAM6-Smad2/3 signaling. This study was the first, to our best knowledge, to explore the important role of TRIM9 in bladder cancer and provide a novel prognostic biomarker for clinical early diagnosis.

Previous studies have focused on the oncogenes that drive tumor progression, and explored novel molecular targets for precise treatment in bladder cancer (Xie et al. 2017; Wu et al. 2023; Huang et al. 2015). CEACAM6 is a member of the carcinoembryonic antigen molecule family, which widely distributed in epithelial and myeloid cells (Rizeq et al. 2018). It acts as an intercellular adhesion molecule to organize tissue architecture and regulating different signal transduction by interacting with other surface proteins (Johnson and Mahadevan 2015). The aberrant expression of CEACAM6 leads to the development of human malignancies (Duxbury et al. 2004; Poola et al. 2006). Recently, CEACAM6 has been identified as a prognostic factor of survival and recurrence in many tumors given its involvement on cell differentiation, growth, invasiveness, metastasis and drug resistance (Burgos et al. 2022). For instance, CEACAM6 induces EMT and mediates invasion and metastasis in pancreatic cancer, gastric cancer via PI3K/AKT signaling pathway (Chen et al. 2013; Zang et al. 2014). Other studies identified that CEACAM6 was a factor of independent prognostic significance in colorectal cancer and gastric cancer (Jantscheff et al. 2003; Zhao et al. 2011). Remarkably, this study, for the first time, indicated that CEACAM6 played an important role in the TRIM9 induced bladder cancer progression. Our study found a significantly elevated CEACAM6 expression in TRIM9 high expressed bladder cancer and it was associated with poor survival of patients. Moreover, we illustrated that the inhibition of CEACAM6 by si RNA could reverse the cell proliferation, migration, chemo-resistance induced by TRIM9. Since previous studies have suggested that CEACAM6 could be a potential target for different cancer immunotherapies (Han et al. 2020), our evidences further highlighting its role as both a prognostic and a therapeutic target in bladder cancer.

Previous works revealed that overexpressed CEACAM6 resulted the alteration and reorganization of the extracellular matrix and reestablished a promoting tumor environment by means of signal transduction (Rizeq et al. 2018). CEACAM6 signaling was shown to activate SRC kinase, leading to increased IGF-1 expression, PI3K/AKT pathway activating and MMP-2 elaboration (Sen and Johnson 2011; Hammarström 1999). And the activated SRC-AKT signaling was reported to contribute to chemoresistance in pancreatic ductal adenocarcinoma cell lines (Heinemann et al. 2014). Notably, it was documented that there was an positive loop between CEACAM6 pathway and TGF-β signaling. The SRC-AKT signaling induced MMP2 expression could activate TGF-β/Smad3 signaling, which further promotes the gene expression of CEACAM6 (Rizeq et al. 2018). Meanwhile, it has recently been suggested that the TGF-β/Smad2/3 signaling pathway plays significant roles in cancer metastasis and progression (Batlle and Massagué 2019). Indeed, in our study, we found that the TRIM9 induced CEACAM6 overexpression upregulated the phosphorylation of Smad2/3 in bladder cancer cell lines, and the Smad2/3 inhibitors ITD-1 effectively suppressed the TRIM9 overexpression associated proliferation, metastasis and chemoresistance in vitro and in vivo. In addition, TGF-β induced MMPs expression has been shown to enhance the invasion and migration capabilities of various cancer cells (Zare et al. 2020; Sinpitaksakul et al. 2008; Nannuru et al. 2010), and our study also identified an increased MMP2 expression in the CEACAM6/Smad2/3 signaling medicated bladder cancer progression. Our results revealed the molecular mechanisms by which TRIM9 facilitates tumor growth, metastasis and chemoresistance in bladder cancer, and provided a promising combined therapy strategy to improve the outcome of chemotherapy.

This study initially investigated the TRIM9 regulation of bladder tumorigenesis and explored its underlying mechanism. First, the transcriptional analysis of the TCGA database indicated that TRIM9 was associated with high clinicopathological stage and poor survival in bladder cancer patients. The same results were confirmed in our clinical samples. Secondly, our exploration about effects of TRIM9 on cell biological processes showed that TRIM9 promoted bladder cancer proliferation, migration and chemoresistance via CEACAM6/Smad2/3 signaling. Then, we established TRIM9 overexpressed bladder cancer mice model, verified the pro-tumor function in vivo, and suggested that the blockage of Smad2/3 signaling significantly improved the chemotherapy outcome in bladder cancer, which highlighting the potential clinical significance of TRIM9/Smad2/3 signaling in bladder cancer. However, there were still some limitations in our research. The included patient samples for clinical pathological evidence were limited, which made the clinic relevant power limited at this point. In addition, TRIM9 has been identified as E3 ubiquitin ligase, thus the relationship between TRIM9 and the CEACAM6 signaling may be involved with ubiquitination, epigenetic modification, and crosstalk with other pathways, which inspires more in-depth researches in future. And the specific regulatory mechanism of CEACAM6-mediated, aberrant activation of Smad2/3 signaling remain to be elucidated in further investigations.

## Conclusion

In summary, our experiments suggest that TRIM9 plays an oncogenic role in bladder cancer progression by increasing cell proliferation, migration, and inducing chemoresistance via CEACAM6/Smad2/3 signaling pathway. Moreover, the combination of Smad2/3 signaling inhibitor and chemotherapeutic agents effectively improved the treatment outcome of bladder cancer *in vivo*.

Thus, the TRIM9/Smad2/3 signaling might represent promising prognostic markers and therapeutic targets for bladder cancer.

### Electronic supplementary material

Below is the link to the electronic supplementary material.

Supplementary Material 1

## Data Availability

The public data were downloaded via cbioportal (https://www.cbioportal.org/).

## Declarations

### Registry and the Registration No. of the study

Ethics Committee Approval No. 2022-028.

### Animal Studies

All animal experiments are performed and approved according to the guidelines of the Ethics Committee of Qilu Hospital of Shandong University.

## Abbreviations

TRIM: Tripartite motif containing
TCGA: The Cancer Genome Atlas
MMC: Mitomycin C
GEM: Gemcitabine
CEACAM: Carcinoembryonic antigen
MMP: Matrix metalloproteinase
GO: Gene Ontology
KEGG: Kyoto Encyclopedia of Genes and Genomes
ELISA: Enzyme linked immunosorbent assay
