# Supplementary material for: Tripartite motif–containing 9 promoted proliferation and migration of bladder cancer cells through CEACAM6-Smad2/3 axis
Source: J Cell Commun Signal. 2023 May 30;17(4):1323–33. doi: 10.1007/s12079-023-00766-7 (PMC10713968; doi:10.1007/s12079-023-00766-7)
Supplement: Supplementary file 1 — Supplementary Material 1 [file 12079_2023_766_MOESM1_ESM.docx]

**Supplementary information**

Supplementary figure 1


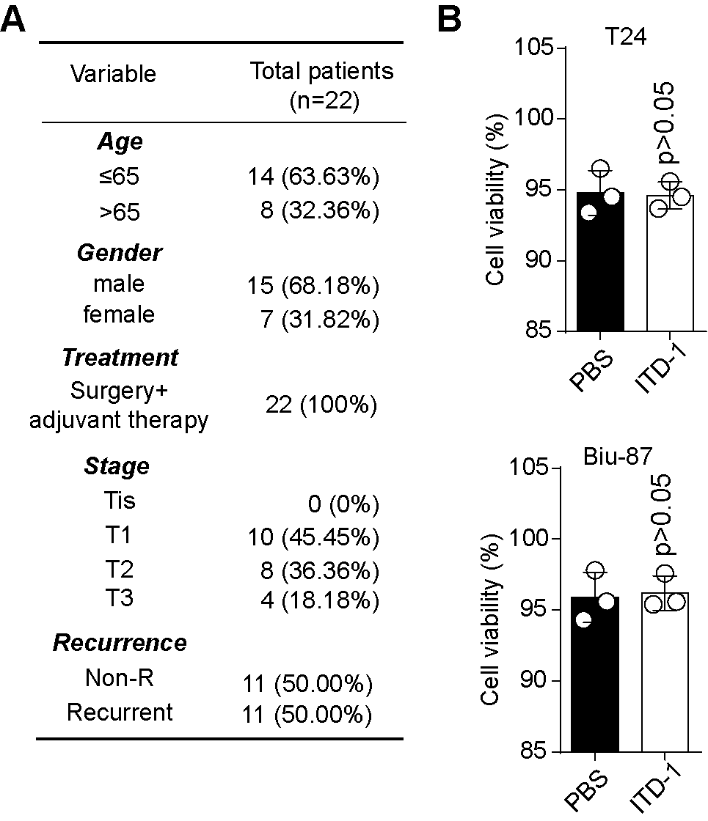


A, information of 22 clinical patients with bladder cancer. B, TRIM9 overexpressed T24/Biu-87 cells treated with PBS or ITD-1 (30 nM). Cell apoptosis was then determined.
